# "I did not intend to stop. I just could not stand cigarettes any more." A qualitative interview study of smoking cessation among the elderly

**DOI:** 10.1186/1471-2296-12-42

**Published:** 2011-05-31

**Authors:** Astri Medbø, Hasse Melbye, Carl Edvard Rudebeck

**Affiliations:** 1 Institute of Community Medicine, University of Tromsø, 9037 Tromsø, Norway

**Keywords:** Decisions, general medical practice, qualitative, smoking cessation, spouse

## Abstract

**Background:**

Every year, more than 650,000 Europeans die because they smoke. Smoking is considered to be the single most preventable factor influencing health. General practitioners (GP) are encouraged to advise on smoking cessation at all suitable consultations. Unsolicited advice from GPs results in one of 40-60 smokers stopping smoking. Smoking cessation advice has traditionally been given on an individual basis. Our aim was to gain insights that may help general practitioners understand why people smoke, and why smokers stop and then remain quitting and, from this, to find fruitful approaches to the dialogue about stopping smoking.

**Methods:**

Interviews with 18 elderly smokers and ex-smokers about their smoking and decisions to smoke or quit were analysed with qualitative content analysis across narratives. A narrative perspective was applied.

**Results:**

Six stages in the smoking story emerged, from the start of smoking, where friends had a huge influence, until maintenance of the possible cessation. The informants were influenced by "all the others" at all stages. Spouses had vital influence in stopping, relapses and continued smoking. The majority of quitters had stopped by themselves without medication, and had kept the tobacco handy for 3-6 months. Often smoking cessation seemed to happen unplanned, though sometimes it was planned. With an increasingly negative social attitude towards smoking, the informants became more aware of the risks of smoking.

**Conclusion:**

"All the others" is a clue in the smoking story. For smoking cessation, it is essential to be aware of the influence of friends and family members, especially a spouse. People may stop smoking unplanned, even when motivation is not obvious. Information from the community and from doctors on the negative aspects of smoking should continue. Eliciting life-long smoking narratives may open up for a fruitful dialogue, as well as prompting reflection about smoking and adding to the motivation to stop.

## 1. Background

Cigarettes are used to handle stress, to relax and to comfort, or as a reward. People socialize, deal with difficulties and have a good time through smoking [1]. People also achieve group membership and status, and develop their identity through smoking [2].

Smoking is considered to be the single most preventable factor influencing health [3]. More than 650000 Europeans die every year because they smoke [4]. Over the past 25 years, smoking has decreased in Western Europe [5]. More than 70% of smokers in the western world want to quit, but only 2-3% do so permanently each year [3,6-9] The governments in Europe and the USA expect general practitioners (GPs) to provide health information, and GPs are encouraged to use an interventional model called "The five As": *ask, advise, assess, assist *and *arrange *for every smoker at every visit to map out smoking [3,6-8]. The five As invite patients to a 2-5 minute person-to-person conversation on smoking [3,6-8,10]. After minimal intervention by health providers some 1-3% of smokers will stop [9,11]. Nicotine replacement therapy (NRT) is recommended [6-8,10], and improves the cessation rate by an odds ratio of 1.77 [8].

Motivational interviewing (MI) aims to explore the patient's ambivalence about change, and to encourage the patient to make her or his own decisions on how to proceed [6,7,12]. A recent meta-analysis showed MI to result in smoking cessation at a slightly higher rate than "brief advice" and "usual care" [13]. As a brief model aimed at opportunistic intervention, MI was judged applicable in usual practice by GPs [12] even though, being a "brief " intervention it made the consultations longer [6]!

The usefulness of standardised and structural programs has been questioned. Ritchie et al [14], Aveyard et al [15] and Riemsma et al [16] found that such programs were insufficient for many smokers. In addition only a minority of GPs seem to advise their smoking patients about stopping [17]. One reason for their reluctance to intervene was given in a study by Guassora et al [18]: the interviewed GPs said more problems were produced than solved by discussing smoking cessation in "every" GP consultation. If the GP gives advice not consonant with what the patient expects, trust can be strained [19]. There are also practical obstacles: it takes time to assess motivation, and doctors feel they lack the training [6,20]

In a population-based survey from Tromsø, a city in Northern Norway with 61.000 inhabitants in 2001, about 82% of the men and 53% of the women 60 years and older had once been daily smokers, and 23% of both sexes were so still [21].

In view of the moderate effects of such an elaborate conversation strategy as MI, the high quitting-rate in the Tromsø-population, made us curious. What had happened? How and why did they do it? Smoking among teenagers, pregnant women and young adults is well described in the literature [8,22,23], but there are few qualitative studies of smoking among the elderly, who represent a substantial proportion of the Tromsø decrease [21].

Polkinghorne [24] claims that stories are the linguistic form in which lived human experience may be suitably expressed. We wanted to talk to elderly smokers and ex-smokers to learn what made them quit or go on smoking. With the emphasis on the elderly, our aim was to gain insights that may help general practitioners to understand why people smoke, and why smokers stop and then maintain quitted; and from this to find fruitful approaches to the dialogue about smoking cessation.

## 2. Methods

Qualitative content analysis (QCA) across narratives was used. Regarding QCA, the method refers to Graneheim & Lundman [25] and Elo & Kyngnäs [26]. The narrative approach applied has been described by Frank [27-29].

### Participants

Population-based studies have been conducted in Tromsø since 1979. The sixth Tromsø study was conducted in 2008, and the present informants were recruited from that survey. The study is part of a bigger project examining persons of 60 years and older. We planned to invite 15 persons, women and men, current and previous smokers. Many current smokers have stopped but relapsed; and to understand successful cessation better, we wanted the contrast given by those who failed to maintain quitting. Randomly selected within sex, smoking status, and age group, individuals were mailed an invitation to talk, in their own homes, about their experience of being a smoker/ex-smoker. The letters were sent consecutively to 57 persons until we had five smokers. In all 20 persons had then responded positively. Two of eleven women smokers answered positively (one aged 58), three of thirteen men smokers, three of sixteen women ex-smokers and twelve of seventeen men ex-smokers (Table 1). We lost contact with two of the latter, and thus 18 were interviewed. We sent no reminders to the non-responders. It turned out that all the smokers had also made temporary stops.

**Table 1:** Characteristics of the informants.

Informant number, sex and name *	Age	Status Ex = ex-smoker S = smoker	Age at start	Years as smoker	Maximum number of cigarettes a day	Serious diseases	Years since the last stop
1. Male Anders	77	Ex	15	48	15	2 myocardial infarctions, angina, bypass, chr. bronchitis,	14

2. Male Jon	64	S	14	> 50	50	2 myocardial infarctions, angina, bypass, aorta aneurysm	

3. Male Kristian	72	Ex	18**	> 20	40	Angina, bypass	34

4. Female Lilly	66	Ex	18	40	20	Bladder cancer, COPD****	8

5. Female Anna	68	Ex	18	46	10	Stomach ulcer, asthma.	4

6. Male Paul	63	Ex	15	10	15-20		38

7. Male Petter	72	Ex	14	42	50	3 strokes	16

8. Female Mary	62	Ex	13	40	10		9

9. Male Edvard	68	Ex	13	40	30		15

10. Male Georg	73	Ex	13	60	50	Atrial fibrillation, pacemaker.	<1

11. Male Karl	80	Ex	17	23	10		40

12. Male Thomas	64	Ex	14	44	20	Chr. bronchitis, Aorta aneurysm	6

13. Male Oscar	65	Ex	13	42***	20	COPD	2

14. Male Sverre	77	Ex	13	17	10		46

15. Male Hans	61	S	15	> 47	15		

16. Female Elisabeth	77	S	17	> 60	10	TIA*****	

17. Male Theodor	61	S	24	35	5-10		

18. Female Nina	58	S	18	> 40	15	Severe pneumonia	

* Pseudonyms by the first author

**First cigarettes about 10 years old

*** Stopped temperately for 8 years

**** Chronic obstructive Pulmonary Disease

***** Transitory Ischemic Attack

Ethical approval was given by the Regional Committee for Medical and Health Research Ethics in Northern Norway. A licence was obtained from the Norwegian Social Science Data Services. All the participants gave written informed consent. They were not paid.

### The interview

An interview guide (see Additional file 1) was constructed to address the research question, and adjusted after a pilot interview with an ex-smoking GP colleague.

The semi-structured interviews were carried out in the participants' homes by the first author, an experienced GP, following the guide. Conversation in the interviews went back and forth until all items were covered. Irrespective of the order of the items, a chronology had been established when the interview ended. The interviews lasted for 20-40 minutes, were audio-taped, and then transcribed verbatim by the first author and an experienced GP colleague. All the interviews were completed between June 2008 and January 2009 before we started the detailed analysis. The quotations were translated into English by the first author.

### Data analysis

The main author wrote her first reflections shortly after each interview. Through this, salient impressions and learning points from the individual interviews were preserved to contribute to the interpretations in the succeeding steps of the analysis. Then, the interviews were read through and listened to several times by the first author to obtain a sense of the whole and to become familiar with the individual interviews. At this stage, the third author also took part in the reading. We put Frank's three narrative questions [29] to the interviews: What does the story do *for *me (the researcher)? What does the story do *to *me? What does the story do *for *the informant? In response to the questions we wrote short summaries and formulated preliminary narrative lines, such as "all the others", "strive" "health implications". We tried to discern different types of major smoking narrative, and we saw that cessation in some cases appeared quite unplanned (see Result). However, the results so far were general and incomplete, and to incorporate the richness of the material systematically, we applied QCA, which is "content sensitive" and "flexible in terms of research design", and can be applied in a defined theoretical perspective [26]. From the perspective of the initial narrative analysis, meaning units describing influences, experience, considerations, decisions and actions concerning continuing or stopping smoking were identified, condensed and coded to express a more general understanding of them (Table 2). Although in one sense decontextualised, the meaning units and codes kept their narrative character. When condensed, each informant's meaning units together constituted a coherent synopsis of the narrative. Based on the codes, sub-themes and themes were developed, and from these, a collective smoking narrative. All the headings of themes and sub-themes were the products of the research, although in one case a sub-theme was identical with a phrase - "all the others" - given by the informants. Because the phrase was very telling, we used it with generalising intent.

**Table 2:** Example of meaning units, condensed meaning units, interpretation and themes, from content analysis.

Meaning Unit	**Condensed Meaning Unit Description close to the text**.	Interpretation (Code)	Sub-theme	Theme
"My partner got very sick. He was a heavy smoker. At the hospital they told him to stop smoking. While he was in hospital I decided to stop. I stopped that day and have not smoked since. I knew he would not manage to stop if I continued."	She stopped smoking to help her partner.	Knowing her smoking influences partner's smoking	- Unplanned cessation - All the others	- The cessation act - Aware of the drawbacks of smoking
"I really want to stop, but it is so hard. I am willing to quit, but then I find that a cigarette is good for me. It is so cosy. I sit here alone. My cigarette is a little friend."	He knows smoking is harmful, but is addicted and has comfort in the cigarette	Reason fighting well-being	- Ambivalence	- Approaching the cessation decision

There was an ongoing dialogue between the first and third author throughout the analysis. The second author participated in the design and in the writing.

## 3. Results

The stages of the emerging collective smoking narrative were largely both time-based and thematic. For instance, starting smoking was the evident first stage of smoking, but in its thematic sense it was about the drives that made the start happen. Thematically, the stages slightly overlapped, but each had its separate significations that remained relevant for the rest of the smoking career. The stages were "Becoming a smoker", "Smoking becoming a habit", "Growing aware of the drawbacks of smoking", "Approaching a decision to stop", "The actual stopping", and "Maintain quitting". *Sub-themes *that linked the stages with concrete situations are shown in italics in the text when presented for the first time, and are illustrated with quotes. In these, the informants are given common Norwegian names, none of which were the informants' real names.//: indicates that some sentences were left out, and .......that the patient was searching for the right words.

### 3.1. Becoming a smoker

The start of smoking was prompted mainly by external influences. A phrase used was *"all the others"*, referring to friends, sometimes siblings too. The informants were drawn into smoking by their friends, and they expressed no responsibility of their own. To be a member of the group and do what others do fosters conformity and confidence. For some it was socially accepted to start smoking as an adolescent after primary school, others sought the excitement of doing something considered wrong. It was all about fitting into a group, and seeking one's identity as grown-up and cool. Almost all the informants started to smoke at 13-18 because "all the others" did so (Table 1). Smoking was also a family pattern, which the informants seemed to follow without much consideration. None was the only smoker in the family.

Mary (8), 62-year old ex-smoker:

"I started when I was 13. I had some older friends and cousins who tempted me. It was awful. I did not smoke much the first two years, but later it increased. When I was 15 I left home and started to work."

Hans (15), 61-year old smoker:

"Both my parents smoked. I don't think they had any influence, but all my brothers and sisters smoked. We were 7 children. I am the youngest."

*Lack of information *on the risks of smoking was a repetitive theme through many of the smoking stories. The informants seemed to start smoking without knowing or thinking of any negative consequences.

Elisabeth (16) 77-year old smoker:

"Everybody did it, and nobody told us it was dangerous."

### 3.2. Smoking becoming a habit

When one had been a smoker for some time, internal driving forces became increasingly strong. Most of the positive experience from the starting phase was still relevant. Smoking and the social life around the cigarette were enjoyed in their own right. Body and mind got adapted to smoking, and new benefits were experienced. "All the others" were still influential, such as when neighbours or friends met over a cigarette. They smoked at work and when doing business too.

Anders (1) 77-year old ex-smoker:

"Earlier you were almost forced to smoke. In my job I travelled a lot to attend meetings. There were lots of cigarettes and cigars, whiskey and cognac and stuff like that too."

The cigarette became a *habit*. Smoking was the first thing in the morning, and was continued whenever possible; in the car, at the office or after dinner. The informants relaxed, handled stress and mixed with friends by smoking.

Georg (10), 73-year old ex-smoker:

"After a while I smoked even more. At the end I rolled a cigarette while driving. I smoked a pack (50) a day. I did so for years!"

Theodor (17) 61-year old smoker:

"....If I deal with a problem which I cannot solve I put the problem aside for a time, and light a cigarette. Then I feel more calm and even-tempered and feel a bit...I have experienced it many times: stress and a cigarette. They should probably not be combined,... but I grow less stressed."

### 3.3. Growing aware of the drawbacks of smoking

*Information *on the damage to health caused by smoking from doctors and other sources, efforts by society through new legislations on tobacco restrictions, and rising prices, made the informants aware of the negative sides of smoking.

Hans (15) 61-year old smoker about the price of tobacco:

"It's fairly expensive to smoke... two packets/tins of tobacco including paper cost 380 NOK a week, times 52 makes 20.000 NOK a year...We could travel to the south every year...."

The implication of awareness was, however, that for some time the unpleasant messages of smoking did not really go home. The informants had a vague feeling of doing something wrong, but still enjoyed smoking. Some remained aware only briefly, others for years. Although not prepared to stop, all the smokers had told their GPs about their smoking, and they appreciated the GP's involvement.

Jon (2), 64-year old smoker:

"My GP often comments on my smoking. I bring his comments back home and think them over. But... I find: I'm 64, and starting to push myself now... "

Among "all the others" were close relatives, who by being worried about the informants' smoking, evoked awareness, as did the smokers' care for spouses, children and grandchildren.

Jon (2), 64-year old smoker:

"When our grandchildren visit us we open the doors and go outside for a cigarette. //...They should have been here always...."

*Threatening signals from the body *and recognizing *abstinence *evoked awareness too. Some gradually grew in awareness as young adults, others after many years as smokers. Some became aware through the weak signals of breathlessness during exercise, while others had to suffer serious diseases before realization dawned that smoking did harm.

Jon (2), 64-year old smoker:

"My wife tried to scare me about my heart. I already knew in 89 when I had my two first infarctions that the cigarettes contributed, but I disregarded this."

Kristian (3) 72-year old ex-smoker:

"I did not smoke all day until I came home from work. One hour before I left work I felt the craving. I had to hurry home. I came home to my children. The first thing I did was to roll two cigarettes and smoke them before eating. I smoked all evening, even more than I would have done if I had smoked at work...It still bothers me...."

### 3.4. Approaching a decision to stop

Sooner or later, awareness demanded *reflection *on the consequences of smoking, and most of the informants started to consider quitting. Awareness was dealt with, and motivation towards stopping was growing. The enjoyment of cigarettes and awareness of the negative sides of smoking from earlier phases were still present, but the thoughts of stopping were uppermost in the informants' minds. For those who quitted smoking, reflection grounded a motivation strong enough to end up in a decision.

Kristian (3), 72-year-old ex-smoker about his last stop:

"I had been working on my decision, you can say.// I did not dread the stop because if it turned out to be too hard I would start smoking again. I considered...my economy and how much I smoked...this influenced my motivation to stop."

The stated reason to stop was almost always *health-related*. Increasing knowledge and the recognition of health improvement increased determination. Most smokers also feared to harm spouses, children and grandchildren.

Also, the influence of "all the others" was active when approaching a decision:

Theodor (17), 61-year-old smoker:

"My spouse smokes. She has some influence on me. I have told her: "If you stop smoking, I will stop too".

Hans (15) 61-year-old smoker about what could motivate him to stop:

"No.. to protect my wife ...or if I cannot walk in the mountains anymore or if I get sick, COPD for instance."

For some, the *struggle *and the *fear of craving *hindered them from stopping.

Karl (11) 80-year old ex-smoker:

"I did try to stop many times before, but I threw my tobacco into the garbage. Now I've stopped! Some minutes later I panicked and ran to the nearest newsagent's and bought new tobacco. You get panic-stricken when you are without tobacco. This feeling, I can still feel it, the panic."

The process towards stopping might also be slowed down or stopped in *ambivalence*. Emotionally, dependence, habit and enjoyment counteracted a decision to stop. Some never raised enough motivation to stop. They were aware of the negative sides of smoking, had reflected somewhat, felt the ambivalence but stayed smokers.

Jon (2), 64-year-old smoker:

"I really want to stop, but it is so hard. I am willing to quit, but then I find that a cigarette is good for me. It is so cosy. I sit here alone. My cigarette is a little friend."

When the reflection had lasted for some time, the ambivalence had moved back and forth, it hardened into *resolution*, and the smoker waited for an appropriate opportunity to stop.

Edvard (9), 68-year old ex-smoker had for years been thinking of stopping because of his daughters' asthma. Suddenly he decided:

"I went to my doctor and then I got this smoking plaster. I decided: It is enough! I have not craved or wanted a cigarette since then. It was so strange and different from the other times I've stopped.// I used the plaster one or two weeks. I do not think it was the plaster, but that this time I really had *decided *to stop: I was motivated."

### 3.5. The actual stopping

Some of the informants had moved through all the stages, some for a few years, others for many, before quitting. Others seemed to skip, or just touch, the awareness and/or motivation stages before they actually stopped smoking. None of the 13 who had stopped did so in response to concrete advice from a doctor.

Nine informants seemed to have stopped suddenly and *unplanned*. The informants seemed to slide into a life as a non smoker without a clear decision, or a clear insight into what harm the tobacco had done to them. They stopped without visible internal struggle or resolution, and therefore it was only the stopping itself that could distinguish the stoppers from those who continued. Without accidental circumstances such as acute bronchitis, a sick family member or acute heart diseases, they would probably have continued. In these cases no hard decisions had to be made, no harm from smoking was felt and the struggle following the stop was little.

Anna (5), 68-year old ex-smoker:

"I had bronchitis twice in a row. Cigarettes did not taste any more. I did not decide to stop, but it didn't taste. And I stopped smoking, and have not been smoking since."

Lilly (4), 66-year old ex-smoker:

"My partner got very sick. He was a heavy smoker. At the hospital they told him to stop smoking. While he was there I decided to stop. I stopped that day and have not smoked ever since. I knew he would not manage stopping if I continued smoking."

Four informants had acted *resolutely*: They felt that tobacco reduced their physical capacity when training. They had stopped many years previously. One did it easily, but three struggled heavily to succeed. Reflection, motivation, physical awareness and addiction were important issues when stopping was resolute.

Sverre (14), 77-year old ex-smoker:

"I realised when playing football and handball that the ones who did not smoke breathed more easily than me. Sometimes I had almost no breath.// I smoked for about 15 years, until 1963. I did not smoke so much, and decided to stop. I had a hard time, but I used Menthol pastilles."

### 3.6. Maintaining quitting

Suddenly or finally 13 of the informants had stopped smoking. To avoid relapse, some had prepared for the craving and the restless fingers, and for certain situations such as parties involving alcohol. Only two used medication to manage to stop, and both just for 1-2 weeks. The others stopped on their own.

All but one *kept tobacco handy *in their pockets or at home for 3-6 months after quitting.

Karl (11), 80-year old ex-smoker:

"I tried to stop smoking many times before, but I did some silly things. I threw the tobacco away. But then I found out: I should have all my pockets full of tobacco, cigarettes and matches and everything you need. When I felt the craving I touched my pockets: "I have it right here! I can wait a bit longer." I continued for three months until I found that I could throw the tobacco away."

Informants who thought they would have to overcome a huge barrier to stop smoking found the passage *easier than expected*.

Thomas (12), 64-year old ex-smoker:

"I stopped five years ago while I was hospitalized for an aorta operation. //Five years have passed without smoking. It's no problem to stay stopped. All it takes is not to start again."

## 4. Discussion and Conclusion

### 4.1. Discussion

We present a smoking narrative from debut in adolescence to cessation about 40-60 years later. "All the others" had a crucial role in all stages of smoking, from the start, via habituation to awareness and the approaching decision, and finally to stopping and sometimes relapsing. Most of the informants stopped smoking unplanned. Nicotine replacement therapy (NRT) was often not considered necessary for success. The majority of smokers believed that keeping tobacco handy helped to maintain their cessation. Information from the community and doctors enhanced awareness of the negative effects of smoking, and may have influenced the motivation for stopping.

#### 4.1.1. Existing models and standardised approaches

The transtheoretical model of change (TTM) of Prochaska et al [30] assesses the willingness to abandon problem behaviour, and hence to give a basis for developing effective interventions to promote change. The TTM contains five phases - pre-contemplation, contemplation, preparation, action and maintenance [30,31]. It has much in common with motivational interviewing (MI), but the two are not parts of the same package [12,13]. Aveyard et al [32] have tested TTM in smoking cessation, and Riemsma et al [16] have systematically reviewed stage-based intervention in smoking cessation. Both found no evidence for effects of TTM. Despite this knowledge TTM is still recommended in smoking cessation [7].

Superficially, the smoking narrative we describe resembles the structure of the TTM, although the latter was not within our theoretical perspective when analysing our material. In contrast with the TTM, the smoking narrative puts smoking in a detailed biographical context, as well as describing and interpreting what goes on before awareness occurs. Starting with contemplation gives too limited an entry into the reality of smoking cessation. In a life perspective, the stages of smoking turn into experience, drives, emotions and arguments in an ongoing process of consideration with very individual patterns and with very strong social influences; and the outcomes of this process are difficult to forecast.

One lesson from our study is that its method of interrogation, i.e. eliciting the life-long smoking narrative, as an ingredient of doctor-patient interaction, may induce awareness and/or motivation by itself, as well as paving the way for a fruitful dialogue. The link between the passing of time and the changes in thinking about one's own smoking has its own explorative dynamic that made up the richness of the material of our study. We believe that this dynamic would be valid also in the consulting room. Therefore we believe it is realistic to see motivational interviewing, even in its brief intervention version, as a second step, when smoking cessation is the agreed reason for the encounter. Depending on the GP's workload and interest, this more extensive motivational work might well be taken over by other members of the team. In a patient-centred perspective, however, a categorical approach such as the 5As would hardly prove appropriate. If the patient's own reason for the encounter is more ore less exchanged for a public agenda of smoking cessation, the common ground and trust-based doctor-patient relationship crucial to patient-centred medicine will never be established [33].

#### 4.1.2. "All the others"

Smoking is a very social activity, and the influence of other people is well known [34,35], but is absent in TTM, MI and the 5 As. Our study indicates that a smoker is influenced by his/her close relations in all stages of smoking and throughout life, not least at the start. Parents are role models for their children in smoking, too [36]. If a parent smokes the likelihood for the child to smoke is increased: trends and subcultures exert heavy influences. Smoking is more common in lower social classes [5,11,34,37]. This also reflects the role of "all the others" among the economically disadvantaged. When people start smoking, the role of "all the others" - parents, friends and conceived cultural codes - still influences young people's smoking [23,34,36], even although beginners are no longer totally ignorant of the health damage of tobacco. It is plausible that referring to "all the others" not only manifested the dependence on others and the wish to adhere to group norms, but sometimes was self-deceit to escape from responsibility. The decision to stop also depended on whether persons close to the smoker decided to stop. Spouses stopped smoking to care for or in companionship with their partners. Christakis et al [38] found the chance that a person stopped smoking increased by 67% if a spouse also stopped, by 36% if friends stopped, and by 25% if siblings did so. Simmons [39] describe the importance of support from the family when stopping after serious diseases. Although a smoker's own responsibility ultimately makes the difference between smoking and non-smoking, the impact of context is very great. Focusing on individual health without taking into account close relations and/or cultural influences is too narrow an approach for many smokers.

#### 4.1.3. Unplanned cessation

Our finding that the act of cessation was either unplanned or resolute may have important implications. The distinction may be somewhat categorical, but our findings indicate that it is possible to stop smoking even at very low levels of motivation. Some of our smokers quitted almost on impulse, in contrast with the thorough preparation and motivation of those who were resolute. It may be that the act was preceded by a more complex but non-articulated decision process. If so, this did not come out in the interviews, and neither did the reason why they had not quitted before in similar situations. Cessation at low levels of motivation has also been described by West and Sohal [40], who, in addition, found that unplanned attempts to quit were more likely to succeed (OR 2.6) for at least six months than planned attempts were. They propose a model of change based on catastrophe theory where small triggers can lead to sudden changes in motivational state.

#### 4.1.4. Strategies for maintaining quitting

The established advice to a person who plans to cease smoking is to remove all tobacco products prior to quitting [41,42] to reduce the temptation. All our informants said they kept tobacco in their homes/pockets for 3-6 months after their final stop. They used this practice as a safety net. We do not extend this finding to a general recommendation to keep tobacco handy when quitting smoking, but the finding does invite further studies about successful smoking cessation. In addition, we believe it takes an open and cooperative mind to seek strategies that are appropriate to the individual patient.

The use of nicotine replacement therapy (NRT) and pharmacotherapy is recommended in smoking-cessation guidelines [3,6,7,7,10]. Only two of our "stoppers" used such treatment, with plaster or varenicline, but only for 1-2 weeks, seemingly as a symbolic act. They both emphasised the *motivation *as essential for succeeding. One reason why our informants did not use tobacco treatments was that most stopped so long ago that such remedies were not available. This was the case for all the resolute quitters. NRT may be useful, and there is evidence that offering it should continue [7,10]; but it is also important to consider that most quit attempts succeeded without it.

#### 4.1.5. Society and information

In the interviews, the lack of information to beginner smokers on the negative sides of smoking was often emphasized. The informants gained insight over time through information from the community new legislation. They also got information from hospital doctors and GPs, and appreciated these initiatives. The effects of such information are well known [3,6-8,10,30,32].

Some smokers are reluctant to stop. When this is clearly stated, it should be respected by doctors. As emphasized by Guassora et al [19], a GP should not put trust on strain.

#### 4.1.6 External validity

Although all our informants were 58 years or older, our findings are still relevant for future smokers. No-one escapes influence from "all the others", and once established as tobacco-users, most smokers will consider stopping and will pass through variations of the stages here described. The major difference from 60 years ago is knowledge of the harmful effects of tobacco. Those who start smoking today are fewer and belong in general to lower social classes, and the sub-cultures prone to smoking have become tougher [23,34]. Young people are aware that smoking is potentially harmful, but disregard this knowledge at least for some time [23,35].

#### 4.1.7. Study strength and limitations

Although smoking is one of the biggest health burdens known, there are rather few qualitative studies on smoking cessation among the elderly. We have found no other studies describing a smoking story from the start as youngsters until the cessation in middle-age or later.

When planning the study we estimated that a sample of around15 smokers and ex-smokers of both sexes would give enough information. Fewer women and current smokers than ex-smoking men responded, but the further inclusion of smokers and women would probably not have altered the general structure of the narrative. The informants may have had a special interest in talking to us about their smoking, which could give a selection bias. Nevertheless, we still consider our sample to be complex enough to catch important aspects of smoking cessation.

Generally, narratives are media through which people position themselves in the world; explore who they are in relation to others [29,43]. They are recipient-designed: a narrative changes with the passing of time, and in relation to the receiver. It is part of a communicative setting where two or more people seek shared understanding of experience and ideas. All our interviewees knew that the interviewer was a GP. They may have given answers they thought she wanted to hear. However, the plenitude of clear statements of low motivation does not support this assumption. The informants' narratives do not represent absolute truth. Facts may have been re-edited as a work of memory. Still, the version given at the moment of our conversation, and as given in a consultation, was valid as a subjective starting point for change.

We believe that doing the interviews in the informant's homes strengthened the method. The informants could be expected to feel more calm, relaxed and free, and invited to present other kinds of story than those usually presented in a GP's surgery [44].

The data were collected over six months and analyzed only when all had been collected; thus inconsistency should not be a problem.

## 4.2. Conclusion

When exploring a smoking patient's thoughts about quitting, GPs need to supplement an individual perspective with a contextual one. "All the others'", especially spouses' roles, are crucial, both as factual influences, and as a possible escape from responsibility. Strategies for cessation should be agreed in the individual case, and unorthodox solutions suggested by patients, such as keeping tobacco handy, need to be discussed. NRT or varenicline are not always necessary for a successful stop, and patient preferences should therefore be explored. We propose eliciting the smoking narrative as a basic approach in general practice, when smoking cessation is relevant; either at the patient's request, or as called for by smoking-related ill health where the patient asks for help. Doing this may open up for a fruitful dialogue, as well as inducing reflection about smoking and adding to the motivation to stop. Some smokers may stop unplanned with little motivation, so that the GP's interest in the smoking narrative may sometimes be enough to encourage cessation.

## Competing interests

The authors declare that they have no competing interests.

## Authors' contributions

AM conceived of the study, planned the design together with CER, did all the interviews, transcribed about half of them and drafted the manuscript. CER also planned the design, read all the interviews, drafted the manuscript and was the main supervisor. HM contributed in all parts from planning the design to the writing process. All the authors read and approved the final manuscript.

## Pre-publication history

The pre-publication history for this paper can be accessed here:

http://www.biomedcentral.com/1471-2296/12/42/prepub

## Supplementary Material

Additional file 1 The interview guide Click here for file

## References

[B1] Jonsdottir R Jonsdottir H The experience of women with advanced chronic obstructive pulmonary disease of repeatedly relapsing to smoking Scand J Caring Sci 2007 21 297 304 10.1111/j.1471-6712.2007.00472.x 17727541

[B2] von Bothmer MI Mattsson B Fridlund B Influences on adolescent smoking behaviour: siblings' smoking and norms in the social environment do matter Health Soc Care Community 2002 10 213 220 10.1046/j.1365-2524.2002.00363.x 12193164

[B3] Sosial-og helsedirektoratet Røykeavvenning i primærhelsetjenesten Nasjonale og faglige retningslinjer 2004 IS-1171

[B4] Sosial-og helsedirektoratet 2010 http://www.tobakk.no

[B5] Franchi EFA Book on Chronic Obstructive Pulmonary Disease in Europe Sharing and Caring 2009 Italy: EFA European Federation of Allergy and Airways Diseases Patients Associations EFA Central Office 1 116

[B6] A Clinical Practice Guideline for Treating Tobacco Use and Dependence: A US Public Health Service Report JAMA 2000 283 24 3244 3254 10.1001/jama.283.24.3244 10866874

[B7] A clinical practice guideline for treating tobacco use and dependence: 2008 update. A U.S. Public Health Service report Am J Prev Med 2008 35 158 176 18617085 10.1016/j.amepre.2008.04.009 PMC4465757

[B8] Aveyard P West R Managing smoking cessation BMJ 2007 335 37 41 10.1136/bmj.39252.591806.47 17615224 PMC1910650

[B9] Sosial-og helsedirektoratet Nasjonal strategi for det tobakksforebyggende arbeidet 2006-2010 2005 I-1112 B

[B10] Claude Lenfant, Nikolai Khaltaev Global initiative for Chronic Obstructive Lung Disease, Global strategy for the diagnosis, managenment, and prevention of chronic obstructive pulmonary disease NHLBI/WHO workshop report 2001 2701 National Institutes of Health, National Heart, Lung, and Blood Institute 2005

[B11] MacIntosh H Coleman T Characteristics and prevalence of hardcore smokers attending UK general practitioners BMC Fam Pract 2006 7 24 10.1186/1471-2296-7-24 16571119 PMC1450291

[B12] Rollnick S Butler CC Stott N Helping smokers make decisions: the enhancement of brief intervention for general medical practice Patient Educ Couns 1997 31 191 203 10.1016/S0738-3991(97)01004-5 9277242

[B13] Lai DT Cahill K Qin Y Tang JL Motivational interviewing for smoking cessation Cochrane Database Syst Rev 2010 CD006936 10.1002/14651858.CD006936.pub2 20091612

[B14] Ritchie D Schulz S Bryce A One size fits all? A process evaluation--the turn of the 'story' in smoking cessation Public Health 2007 121 341 348 10.1016/j.puhe.2006.12.001 17292931

[B15] Aveyard P Griffin C Lawrence T Cheng KK A controlled trial of an expert system and self-help manual intervention based on the stages of change versus standard self-help materials in smoking cessation Addiction 2003 98 345 354 10.1046/j.1360-0443.2003.00302.x 12603234

[B16] Riemsma RP Pattenden J Bridle C Sowden AJ Mather L Watt IS Walker A Systematic review of the effectiveness of stage based interventions to promote smoking cessation BMJ 2003 326 1175 1177 12775617 10.1136/bmj.326.7400.1175 PMC156457

[B17] Jung J Neumann M Wirtz M Ernstmann N Staratschek-Jox A Wolf J Pfaff H Validation of the "SmoCess-GP" instrument - a short patient questionnaire for assessing the smoking cessation activities of general practitioners: a cross-sectional study BMC Fam Pract 2010 11 9 10.1186/1471-2296-11-9 20122143 PMC2825201

[B18] Guassora AD Tulinius AC Keeping morality out and the GP in. Consultations in Danish general practice as a context for smoking cessation advice Patient Educ Couns 2008 73 28 35 10.1016/j.pec.2008.02.020 18406099

[B19] Guassora AD Gannik D Developing and maintaining patients' trust during general practice consultations: the case of smoking cessation advice Patient Educ Couns 2010 78 46 52 10.1016/j.pec.2009.05.003 19505788

[B20] Skevington SM Day R Chisholm A Trueman P How much do doctors use quality of life information in primary care? Testing the trans-theoretical model of behaviour change Qual Life Res 2005 14 911 922 10.1007/s11136-004-3710-6 16041889

[B21] Medbo A Melbye H Lung function testing in the elderly-Can we still use FEV(1)/FVC<70% as a criterion of COPD? Respir Med 2007 101 1097 1105 10.1016/j.rmed.2006.11.019 17239575

[B22] Lumley J Chamberlain C Dowswell T Oliver S Oakley L Watson L Interventions for promoting smoking cessation during pregnancy Cochrane Database Syst Rev 2009 CD001055 10.1002/14651858.CD001055.pub3 PMC4090746 19588322

[B23] Stewart-Knox BJ Sittlington J Rugkasa J Harrisson S Treacy M Abaunza PS Smoking and peer groups: results from a longitudinal qualitative study of young people in Northern Ireland Br J Soc Psychol 2005 44 397 414 10.1348/014466604X18073 16238846

[B24] Polkinghorne D Narrative configuration in qualitative analysis International journal of qualitative studies in education 1995 8 1 5 23 10.1080/0951839950080103

[B25] Graneheim UH Lundman B Qualitative content analysis in nursing research: concepts, procedures and measures to achieve trustworthiness Nurse Educ Today 2004 24 105 112 10.1016/j.nedt.2003.10.001 14769454

[B26] Elo S Kyngas H The qualitative content analysis process J Adv Nurs 2008 62 107 115 10.1111/j.1365-2648.2007.04569.x 18352969

[B27] Frank A What Is Dialocigal Research, and Why Should We Do It? Qualitative Health Research 2005 964 964 974 10.1177/1049732305279078 16093373

[B28] Frank A Health stories as connectors and subjectifiers Health: an interdiciplinary journal 2006 10 4 421 440 10.1177/1363459306067312 16973679

[B29] Frank A Studying what stories *do*, as they act for people, but also *on *people (narrative analysis) Lecture 2009 Narrative analysis and theory, PhD course in Health Sciences

[B30] Woody D DeCristofaro C Carlton BG Smoking cessation readiness: are your patients ready to quit? J Am Acad Nurse Pract 2008 20 407 414 10.1111/j.1745-7599.2008.00344.x 18786015

[B31] Armitage CJ Is there utility in the transtheoretical model? Br J Health Psychol 2009 14 195 210 10.1348/135910708X368991 18922209

[B32] Aveyard P Massey L Parsons A Manaseki S Griffin C The effect of Transtheoretical Model based interventions on smoking cessation Soc Sci Med 2009 68 397 403 10.1016/j.socscimed.2008.10.036 19038483

[B33] Stewart M Reflections on the doctor-patient relationship: from evidence and experience Br J Gen Pract 2005 55 793 801 16212856 PMC1562329

[B34] Aarø L Lund KE Vedøy TF Øverland S Evaluering av myndighetenes samlede innsats for å forebygge tobakksrelaterte sykdommer i perioden 2003-2007 Sirus Rapport 2010 3/2009 1 140

[B35] Scheffels J Stilig eller stigma? En sosiologisk studie om ungdom, røyking og identitet Avhandling for dr.polit-graden edition 2008 Institutt for sosiologi og samfunnsgeografi, Universitetet i Oslo: SIRUS, Statens institutt for rusmiddelforskning 1 100

[B36] Aaro LE Lindbak RL Nygaard OS Hetland J [Use of tobacco among Norwegian pupils in secondary school 1975-2005] Tidsskr Nor Laegeforen 2008 128 1815 1819 18787590

[B37] Sosial-og helsedirektoratet Tall om tobakk 1973 - 2006 Sosial-og helsedirektoratet 2007 IS - 1465. 2007

[B38] Christakis NA Fowler JH The collective dynamics of smoking in a large social network N Engl J Med 2008 358 2249 2258 10.1056/NEJMsa0706154 18499567 PMC2822344

[B39] Simmons VN Litvin EB Patel RD Jacobsen PB McCaffrey JC Bepler G Quinn GP Brandon TH Patient-provider communication and perspectives on smoking cessation and relapse in the oncology setting Patient Educ Couns 2009 77 398 403 10.1016/j.pec.2009.09.024 19846270 PMC2787754

[B40] West R Sohal T "Catastrophic" pathways to smoking cessation: findings from national survey BMJ 2006 332 458 460 10.1136/bmj.38723.573866.AE 16443610 PMC1382540

[B41] Cornuz J Willi C Nonpharmacological smoking cessation interventionin clinical practice 2008 17 (Review 110) European Respiratory Review 187 191

[B42] Gulsvik A Bakke PS Lungesykdommer En basal innføring Book 2004 64 70

[B43] Hyden LC Meuwisse In A Sward H Eliasson-Lappalainen R Jacobsson K Berettelsesforskning. Forskningsmetodik før socialvetare 2008 Stockholm: Natur & Kultur 89 104

[B44] Thagaard T Systematikk og innlevelse Book, Fagbokforlaget 2003

