# Supplementary material for: "I did not intend to stop. I just could not stand cigarettes any more." A qualitative interview study of smoking cessation among the elderly
Source: BMC Fam Pract. 2011 May 31;12:42. doi: 10.1186/1471-2296-12-42 (PMC3132720; doi:10.1186/1471-2296-12-42)
Supplement: Additional file 1 — The interview guide [file 1471-2296-12-42-S1.DOCX]

Author list: Astri Medbø, Hasse Melbye and Carl Edvard Rudebeck
Title  : "I did not intend to stop. I just could not stand cigarettes anymore."
A qualitative interview study of smoking cessation among the elderly.

Journal: BMC Family Practice
MS ID  : 1686620783477866

**Additional file.**

| The interview guide. |
| --- |
| \| Questions: \| \| --- \| \| 1. Could you tell me your smoking story? Why and when you started and so on. 2. Did your parents smoke? 3. What positive experience did you have as a smoker? 4. What negative experience did you have? 5. Did you *think of* stopping? When? Under what circumstances? 6. Did you *try* to stop? Tell me why. 7. Why did you perhaps start again? 8. What challenges are there to succeed in stopping? What do/did you dread most? 9. How can you maintain quitting? 10. Did your quality of life change after stopping? How? 11. Does your doctor know you were/are a smoker? 12. What do you think of the smoking law? \| |
